# Working on Genomic Stability: From the S-Phase to Mitosis

**DOI:** 10.3390/genes11020225

**Published:** 2020-02-20

**Authors:** Sara Ovejero, Avelino Bueno, María P. Sacristán

**Affiliations:** 1 Instituto de Biología Molecular y Celular del Cáncer (IBMCC), Universidad de Salamanca-CSIC, Campus Miguel de Unamuno, 37007 Salamanca, Spain; 2 Institute of Human Genetics, CNRS, University of Montpellier, 34000 Montpellier, France; 3 Department of Biological Hematology, CHU Montpellier, 34295 Montpellier, France; 4 Departamento de Microbiología y Genética, Universidad de Salamanca, Campus Miguel de Unamuno, 37007 Salamanca, Spain

**Keywords:** cell cycle, DNA replication, replication stress, mitosis, chromosome instability, common fragile sites, mitotic DNA synthesis, DNA damage response, ultrafine bridges

## Abstract

Fidelity in chromosome duplication and segregation is indispensable for maintaining genomic stability and the perpetuation of life. Challenges to genome integrity jeopardize cell survival and are at the root of different types of pathologies, such as cancer. The following three main sources of genomic instability exist: DNA damage, replicative stress, and chromosome segregation defects. In response to these challenges, eukaryotic cells have evolved control mechanisms, also known as checkpoint systems, which sense under-replicated or damaged DNA and activate specialized DNA repair machineries. Cells make use of these checkpoints throughout interphase to shield genome integrity before mitosis. Later on, when the cells enter into mitosis, the spindle assembly checkpoint (SAC) is activated and remains active until the chromosomes are properly attached to the spindle apparatus to ensure an equal segregation among daughter cells. All of these processes are tightly interconnected and under strict regulation in the context of the cell division cycle. The chromosomal instability underlying cancer pathogenesis has recently emerged as a major source for understanding the mitotic processes that helps to safeguard genome integrity. Here, we review the special interconnection between the S-phase and mitosis in the presence of under-replicated DNA regions. Furthermore, we discuss what is known about the DNA damage response activated in mitosis that preserves chromosomal integrity.

## 1. Introduction

**Figure 1:**
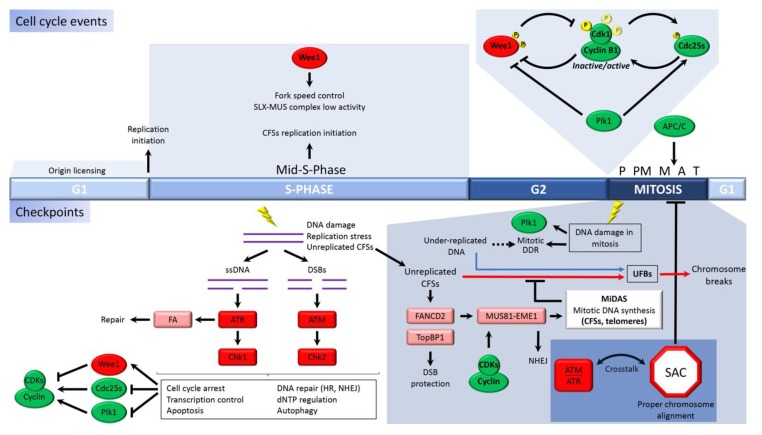
Cell cycle events and checkpoints controlling chromosome stability. Factors promoting cell cycle progression are shown in green, whereas those involved in cell cycle arrest are shown in red and downstream factors of the DDR pathways are shown in pink. The main cell cycle events are represented in the upper part. Origin licensing takes place during G1, and replication initiation is the triggering event of the S-phase. During the S-phase, the kinase Wee1 controls fork speed and maintains the activity of the nuclease complex SLX-MUS (SLX1-SLX4-MUS81-EME1) in a reduced state. CFSs replication initiation starts by the mid-S-phase; this late replication initiation increases the risk of entering into mitosis with under-replicated DNA at these particular loci. At the G2/M transition, the activation of the Cdk1/cyclinB1 complexes is governed by a feedback loop, in which the cyclin-dependent kinase (CDK) complexes inhibit the Wee1 kinase and activate the Cdc25s phosphatases by direct phosphorylation to promote their own activation. The kinase Plk1 contributes to this process by also inhibiting Wee1 and activating Cdc25s. Once in mitosis, the correct attachment to the spindle and alignment of the chromosomes at the metaphase plate satisfies the SAC and allows the activation of the anaphase promoting complex/cyclosome (APC/C). This, in turn, drives the exit from mitosis and guarantees proper chromosome segregation. Checkpoints activated throughout the cell cycle in response to DNA damage or replication problems are depicted in the lower part of the figure. DNA damage in interphase, replication stress, and under-replicated DNA at CFSs activate the ataxia-telangiectasia and Rad3-related and ataxia-telangiectasia mutated (ATR/ATM) pathways and their downstream effectors Chk1/Chk2 in order to arrest cell cycle progression by the inhibition of the CDK complexes and triggering repair mechanisms or programmed cell death, if the damage cannot be repaired. The persistence of incomplete replicated DNA in mitosis activates a DDR, which shares some common effectors with the interphasic mechanisms (ATM, ATR, FANCD2, MUS81-EME1, and TopBP1), in order to induce repair in mitosis or protection of the damaged DNA until the next cell cycle starts and it can be properly repaired. Interactions between DDR and SAC components indicate that the ATM/ATR and SAC checkpoint pathways crosstalk to restrain mitotic progression in the presence of unresolved DNA damage. Moreover, ATM and ATR kinase are involved in SAC regulation in both cells exposed to DNA damage and normal cycling cells. Mitotic DNA synthesis (MiDAS) is the most recently identified mechanism to complete replication at CFSs and telomeres before the end of cell division. MiDAS also plays an unexpected role in the maintenance of chromosome stability. FA, Fanconi anemia; ssDNA, single strand DNA; DSBs, double strand breaks; CFSs, common fragile sites; DDR, DNA damage response; SAC, spindle assembly checkpoint; HR, homologous recombination; NHEJ, non-homologous end joining; UFBs, ultrafine bridges; P, prophase; PM, prometaphase; M, metaphase; A, anaphase; T, telophase.

During every cell division cycle, the genome must be completely and faithfully replicated only once and correctly segregated into each new daughter cell formed in mitosis. Classically, DNA replication has been considered to take place only during the S-phase. However, over the past twenty years, an increasing amount of data have started to challenge this conception, as it is now known that DNA synthesis can extend from the S-phase to mitosis [1]. The cell division cycle is mainly regulated by cyclin-dependent kinase (CDK) activity [2], where two subunits form CDK complexes. These include a cyclin that is periodically synthesized and degraded at specific cell cycle phases and a kinase that is regulated by phosphorylation and interaction with activators and inhibitors. Mammalian cells enter the cell cycle in response to the D-type cyclin expression induced by growth factor signaling pathways. Increased CDK activity drives to G1/S transition and, then, regulates replication processes during the S-phase to complete the bulk of DNA synthesis [3]. After this major DNA synthesis, the Cdk1 and Plk1 kinases are activated at the S/G2 transition and create a switch-like mechanism that eventually triggers mitosis [4]. Coordination between DNA replication completion and cell division is fundamental for cell survival. In this regard, recent works support the idea that DNA replication determines cell cycle progression and length. More precisely, during an unperturbed cell cycle, DNA synthesis signals prevent the premature activation of Cdk1 and Plk1 kinases, and therefore premature entry into mitosis [5]. In addition, new data show that in the presence of replication stress (replication fork slow down, pausing, or instability caused by endogenous or exogenous sources), G2/M transition granules (GMGs), mainly formed by the RNA-binding protein TIAR, accumulate in the nucleus of late G2 and prophase cells. GMGs also contain proteins associated with stalled replication forks and RNA metabolism factors and retain Cdk1 to attenuate its activity and prevent mitotic entry [6]. Subsequent to the S-phase, the G2 phase is characterized by increasing CDK activity and is becoming more important, not only because it allows the precise timing of mitosis through activation of specific cell cycle checkpoints but also because late DNA replicating regions are still able to be replicated during this G2 period in many cells [1,7]. Throughout the S and G2 cell cycle phases, mitotic cyclins start to be expressed, and CDK activity continues to increase [4]. However, Wee1 and related inhibitory kinases still attenuate mitotic CDK activity by direct phosphorylation. In addition, increased Cdc25 phosphatase activity is needed to reverse Wee1 dependent CDK inhibition in order to obtain the high CDK activity level necessary to start mitosis at the proper time (Figure 1). The precise regulation of mitotic CDK complexes at the entry to mitosis has been recently reviewed [8]. Thus, during G2, these positive and negative CDK regulators, whose balance is critical to control high CDK activity and the precise timing of mitosis, are targets of different DNA damage checkpoint pathways that monitor and safeguard the integrity of the genome before reaching the G2/M transition [9,10]. This high phosphorylation activity mediated by CDKs, and other mitotic kinases, such as Plk1, is required for orchestrating mitotic events, such as chromatin condensation, nuclear envelope breakdown, or spindle assembly, as well as ensuring correct cell division [11]. About a decade ago, quantitative phosphoproteomics studies showed the mitotic hyperphosphorylation of more than 1000 proteins, most of them CDK substrates [12], as well as more than 500 substrates of Aurora A, Aurora B, or Plk1 kinases [13]. These data perfectly illustrate the enormous importance of phosphorylation processes, in particular those mediated by CDK, in the control of the G2/M transition. 

The tight regulation of the cell cycle is especially evident during mitosis, wherein potential chromosome mis-segregation, emerging from abnormal mitosis, can lead to genome instability. This, in turn, can cause serious diseases, such as cancer or several hereditary syndromes associated with neurological abnormalities, growth retardation, development malformations, immunodeficiency, and microcephaly, among others (reviewed in [14]). To avoid this possibility, mitotic CDK activity itself induces an essential and inherent checkpoint, the spindle assembly checkpoint or SAC, which ensures proper chromosome attachment to the bipolar spindle to guarantee their faithful segregation (Figure 1) [15,16]. Then, at the metaphase/anaphase transition, CDK activity triggers cyclin destruction through the anaphase promoting complex/cyclosome (APC/C) and, in turn, its own inhibition, which is imperative to exit from mitosis [17]. Accordingly, each cell cycle phase, although rigorously controlled and interconnected with its previous phase, has specific and spatiotemporal limited functions. However, new mitotic processes have recently emerged that highlight the high flexibility of the cell cycle and the intrinsic relationship between the S-phase and mitosis in order to maintain the integrity of the genome. The functional details of how these mechanisms work and how they are regulated is not yet fully understood and constitutes the aim of numerous research works. 

## 2. Replication Stress and Under-Replicated DNA in Mitosis

DNA replication, which mostly takes place during the S-phase, is strictly regulated and coordinated with other cellular processes to ensure that the whole genome is replicated once and only once per cell cycle [18,19]. One important feature for successful replication is the tight control of origin licensing. Replication origin licensing takes place from late mitosis until the end of G1 by loading double MCM2-7 hexamers at origins and is a highly CDK activity-dependent process (Table 1) [20,21,22]; for a recent review on origin licensing see [23]. In this way, at the beginning of the S-phase, MCM hexamers are activated at hundreds of thousands of origins distributed in a non-random way throughout the genome forming the ring of the replicative helicase that unwinds the DNA ahead of DNA polymerase [20,24,25,26]. It has been proposed that S-phase initiation is inhibited in many cells until enough origins are licensed by a p53-dependent “origin licensing checkpoint” [26,27,28,29]. Thus, the depletion of origin licensing proteins, such as Cdt1 or Cdc6, in mammalian cells prevents origin licensing and Cdk2 activation and causes a delay in the S-phase onset in non-transformed mammalian cells. By contrast, p53 defective cancer cells depleted for these proteins progress in the cell cycle and accumulate DNA damage [28]. It should also be noted that Cdt1 and Cdc6 are regulated by CDK activity (Table 1). In addition, mouse models deficient for origin activation or usage, due to MCM mutation or downregulation, display embryonic lethality and early cancer onset, which is also related to the status of p53. This highlights the importance of the coordination between origin licensing and the start of the S-phase for genomic stability [30,31,32]. Surprisingly, using non-transformed cells under in vitro culture conditions, recent work has reported that the licensing checkpoint is almost completely inactive in the first G1 after cell cycle re-entry from quiescence/G0. As a result, cells are unprotected from premature first S-phase entry, which in turn increases the risk of experiencing genome instability. Moreover, inducing G1 extension by inhibiting Cdk2 activity increases the time for MCM loading, thus, improving the impaired licensing checkpoint [33]. In conclusion, defects in origin licensing can lead to DNA under-replication, which constitutes a major source of genomic instability [29,34].

Under normal conditions, the replication machinery successfully covers both genomic length and sequence complexity within the S-phase timing. However, cells are frequently exposed to certain anomalous conditions that hinder DNA replication and can lead to DNA replication stress and the potential inability to complete DNA synthesis in time. The term “DNA replication stress” refers to any condition that causes the replication fork progression to slow down, producing fork pausing or even increased fork instability, which leads to unfinished DNA replication and the formation of anomalous replication intermediate structures, chromosome breakage in mitosis, and potential tumorigenicity [86,87]. DNA replication stress can be induced by different sources, for example, nucleotide pool imbalance or shortage, transcription-replication conflicts, anomalous DNA conformation, difficult-to-replicate loci, or DNA damage caused by endogenous cellular metabolism products, such as reactive oxygen species, exogenous chemical compounds, and UV light and gamma irradiation [88,89,90]. Replication stress is greater in tumor cells, where it is generally caused by either the loss of function of some tumor suppressor genes or deregulated oncogene expression [91]. 

The following two main checkpoint mechanisms governed by apical serine/threonine kinases control replication stress and DNA damage response (DDR): ATM (ataxia-telangiectasia mutated) and ATR (ataxia-telangiectasia and Rad3-related). Both of these coordinate cell cycle progression and DNA repair mechanisms to maintain genome integrity (Figure 1). From a simple point of view, ATM associates with DNA double strand breaks (DSBs), whereas ATR kinase, considered to be a master conductor of cellular responses to DNA replication stress, is recruited to single-strand DNA (ssDNA) (see [92] for details) [93,94,95,96]. It is known, however, that ATR and ATM regulation is dependent on each other under certain DNA damaging conditions [92,97,98,99,100,101,102]. Once activated, these apical kinases phosphorylate through specific protein adaptors, downstream checkpoint kinases (Chk1 and Chk2), and an extensive network of substrates that mediate hallmark cellular responses, such as cell cycle arrest, transcription control, dNTP regulation, replication fork protection, inhibition of DNA replication, initiation of apoptosis, autophagy, and DNA repair [103,104,105,106,107]. Although it is now clear that damaged DNA is sensed by checkpoint kinases that connect to DNA repair pathways, the precise mechanisms by which these kinases regulate their multiple substrates are not completely understood and represent an important subject of study in the field.

One of the best characterized functions of DNA damage signaling kinases is cell cycle arrest, which prevents entry into mitosis with damaged DNA. Checkpoint signaling inhibits positive CDK regulators, Cdc25 phosphatases, and activates Wee1, the main kinase responsible for inhibiting mitotic CDK activity [108,109,110,111]. Other mitotic kinases, such as polo-like kinase (Plk1), are also inhibited by DNA damage signaling upon checkpoint activation [112] (Figure 1). Moreover, the tumor suppressor p53 is directly stabilized, inducing a complex transcriptional response (see [113] for details). Thus, the p53-mediated expression of the CDK inhibitor p21 directly contributes to blocking progression through the cell cycle [114]. Cell cycle arrest facilitates most of the mentioned core outputs that impact DNA damage, with DNA repair being one of the final outcomes. ATM and ATR regulate, through direct or indirect phosphorylation, a large set of factors involved in repair processes [115]. Thus, during interphase, the DDR activated through these signaling kinases plays an important role in the maintenance of chromosome stability by avoiding entry into mitosis with under-replicated or damaged chromosomes.

### 2.1. Common Fragile Sites

Some specific genome regions have been found to be particularly difficult to replicate. Among these, the best characterized are telomeres, ribosomal DNA, and common fragile sites (CFSs) [90,116]. All these regions are densely packaged into chromatin, contain repetitive sequences that tend to form DNA secondary structures, lie within actively transcribed regions, and are associated with tightly bound proteins. These intrinsic features hinder replication fork progression through these loci, which then become effective replication barriers, particularly during replication stress, and can lead to entry into mitosis with under-replicated DNA [89,117]. Eventually, in mitosis, they can evolve into ultrafine anaphase bridges (UFBs) connecting sister chromatids, which can finally lead to chromosomal instability. In the following sections, we focus on CFSs as an example of the extraordinary processes that cells carry out in mitosis to resolve problems that originated during the previous S-phase, in order to maintain genomic stability. Thus, CFSs demonstrate the high flexibility of the cell cycle and the close relationship between the S-phase and mitosis to preserve cell integrity. Nevertheless, it is worth noting that there is another type of fragile chromosomal sites, namely early replicating fragile sites or ERFSs, which have some molecular characteristics different from those of CFSs. ERFSs present a high number of replication origins, have an open chromatin configuration, possess a high GC content and, as their own name indicates, are replicated in the early S-phase and arise from a fork collapse during this moment of the cell cycle [118].

In contrast to ERFSs, CFSs are widely considered to be the last regions of the human genome to be replicated [119], are hotspots of genomic instability highly conserved during mammalian evolution, and are associated with the chromosomal rearrangements typical of early stages of human cancers [86,120]. CFSs were first described as sites on human metaphase chromosomes that are particularly prone to forming breakpoints or gaps [121]. This phenomenon is termed “CFSs expression” and requires the activity of endonucleases that process late replication intermediates persisting at CFSs up to mitosis (this mechanism will be described later on). Although no single molecular feature accounts for the fragility of CFSs, several properties have been identified. One of the basic properties that makes CFSs sites inherently unstable is the presence of repetitive sequences. Some of these sequences present AT-rich regions that can form complex secondary structures, which make replication difficult where fork stalling can prevail [122,123]. Moreover, CFSs lie within large and actively transcribed genes, where replication–transcription collision events are inevitable. Thus, the consequent formation of pathological DNA-RNA hybrid structures can induce CFSs breakage, which is more likely in mitosis [124,125]. Recent work on yeast suggests that CFSs not only present an increased tendency to break but also a reduced ability for DNA breaks to be repaired, which instead persist until mitosis [126]. Another characteristic is that CFSs have a low number of replication origins to cover long distances during the S-phase. This origin paucity implies that virtually all the origins present at CFSs are activated under normal conditions. This implies that no dormant origins are available under replication stress, leading to unfinished DNA replication and, consequently, to chromosome breakage in mitosis [86,127]. Work on mammalian cells has shown that the origins of the flanking regions of CFSs are fired in the mid-S-phase, which also increases the chances of the finishing G2 phase with incomplete DNA synthesis in the event of replication slow down. It is known that the distribution of the origins and the replication timing vary between different cell types, which would explain the tissue specificity of CFSs and the higher probability of some cells to undergo malignant transformations when replication problems arise [127]. For more insight on fragile site characteristics, see the recent reviews from Glover et al. and Voutsinos et al. [120,128].

Since CFSs compromise replication completion, cells activate checkpoint signaling pathways when they are not fully replicated. It has been shown that ATR kinase plays a major role in maintaining CFSs stability by activating, through its downstream factor Chk1, the cell cycle checkpoint in response to their incomplete replication. Thus, using ATR conditional KO vertebrate cell lines, it has been shown that the absence of ATR during unperturbed cell cycles leads to replication fork progression slow down and increased replication origin activation. As a consequence, cells with incomplete replication prematurely enter into mitosis and divide faster, leading to aberrant chromosome separation, increased UFBs, cytokinesis failure, and cell death. These results suggest that one of the main roles of ATR may be to ensure that the bulk of DNA replication occurs before mitosis onset [129]. Likewise, ATR inhibition also leads to chromosomal aberrations at ERFSs [118]. Therefore, it is clear that replication stress together with defects in ATR signaling exacerbates CFSs expression [130,131,132]. 

Whereas ATR is crucial for the maintenance of CFSs stability, ATM seems to be important to CFSs only when ATR is not present. Specifically, CFSs expression leads to DNA fragmentation, and therefore the presence of DSBs which activate the ATM pathway through Chk1 phosphorylation [100,131]. It has been shown that Chk2 is also activated under CFS expression conditions, although it does not seem to be involved in the maintenance of CFSs stability [132]. 

In addition to the checkpoint master kinases ATR and ATM, other proteins working in replication stress, DSB repair, or interstrand crosslink repair have also been associated with the regulation of CFSs. It has been shown that Fanconi anemia (FA) proteins, particularly FANCD2, play an important role in protecting CFSs from the S-phase to mitosis [133,134,135,136]. FA is a hereditary genetic disorder characterized by congenital abnormalities, progressive bone marrow failure that results in pancytopenia, and a high risk of developing cancer in early life, including leukemia and head and neck squamous cell carcinoma. FA patients present a high frequency of chromosomal aberrations and high toxicity to chemotherapeutic agents. To date, up to 22 FA-linked genes have been described, all of which play different roles in DNA damage repair by homologous recombination (HR), and their deficiency causes increased endogenous replication stress and DNA damage accumulation. At a molecular level, replication fork stalling activates ATR and the recruitment of the FA core complex (FANCA, -B, -C, -E, -F, -G, -L, -M, and accessory factors like FA100) to the DNA lesion. Then, the FA core complex monoubiquitinates the FANCD2-FANCI heterodimer to stabilize it and promote its retention at the stalled forks. The FANCD2-FANCI complex recruits downstream factors, notably the structure-specific endonucleases SLX1-SLX4, XPF-ERCC1, and MUS81-EME1, that promote the repair of DNA lesions through HR [137,138]. Due to deficiencies in protein members of the FA pathway, FA patients present chromosomal breakpoints at CFSs [139]. Moreover, FA deficient cells also present cytokinesis failure and often produce binucleated cells. These findings support the importance of FA proteins for the prevention of chromosome segregation defects and the protection of genome stability [135]. Furthermore, the FA pathway is interconnected with the ATR signaling cascade to guarantee optimal activation in a reciprocal way [140,141]. Both the ATR and FA pathways, together with proteins such as Rad51, Rad52, the endonucleases ERCC1 and MUS81, the BLM (Bloom syndrome protein) helicase, and DNA polymerases, constitute part of a still growing network of proteins responsible for the maintenance of genomic stability at the CFS loci. A recent proteomics study has identified additional proteins associated with CFSs using chromatin immunoprecipitation (ChIP) of FANCD2 coupled to mass spectrometry [142]. Further analysis of these newly identified, but poorly studied, factors will shed light on the processing of CFS expression.

### 2.2. Fallible Checkpoints Lead to Premature Mitosis with Under-Replicated DNA 

In addition to the previously described G2/M checkpoint that impairs CDK activation, inducing cell cycle arrest and preventing entry into mitosis in the presence of DNA damage, other mechanisms avoid mitosis onset with under-replicated DNA, including CFSs. As already mentioned in the Introduction, recent evidence suggests that DNA synthesis avoids entry into mitosis by inhibiting Cdk1 and Plk1 kinases [5,6]. It has also been proposed that cells can sense the end of the S-phase and coordinate it with cell cycle progression through a S/G2 checkpoint. Recent data indicate that ATR controls the transition from the S-phase to G2 to ensure that replication is completed. At this transition, CDK activity is necessary for the phosphorylation and activation of FOXM1, a transcription factor necessary for the initiation of a mitotic transcriptional program. Active ATR coordinates DNA replication with mitosis through the downregulation of this FOXM1 mitotic gene transcriptional program network (also called FOXM1 phosphorylation switch). Therefore, ATR implements a genuine S/G2 checkpoint [66]. However, this checkpoint is not foolproof; work on fission yeast has established that cells are blind to replication and recombination intermediates and that they do not delay entry into mitosis in their presence. In G2, such intermediates are resolved into acentric and capped chromosomes that yield anaphase bridges which break at the end of mitosis [143]. This blindness seems to happen also in human cells, since there is evidence of DNA replication at CFSs during mitosis [144] and of anaphase bridges that suffer a similar fate to the ones described for fission yeast [145]. Replication fork stalling is especially problematic at CFSs due to their scarce origins, which causes cells to progress into G2 without replication completion at these loci [127]. Upon replication problems, cells can restart the stalled replication forks at CFSs to try to finish replication through a HR-dependent mechanism. However, defects in this restart process can happen if the incorrect template is used (non-allelic homologous recombination or NAHR), leading to gross chromosomal rearrangements and subsequent genomic instability [146,147]. In conclusion, checkpoints are not efficient at suppressing CFSs expression in the S- and G2 phases, and, as a result, cells have evolved other mechanisms to preserve genome integrity at these loci through mitosis and beyond. 

## 3. Crosstalk between the S-Phase and Mitosis

Recently, it has been shown that cells, to resolve incompletely replicated DNA structures persisting in late G2 or early mitosis, activate specific DNA synthesis pathways beyond the S-phase to complete genome duplication and avoid chromosome mis-segregation. Thus, unscheduled DNA synthesis has been observed in late G2 and early mitosis [144,148,149,150].

### 3.1. DNA Synthesis of Under-Replicated DNA Tracks Prior to Mitosis

Since the genome must be completely and faithfully replicated only once during each cell cycle, cells have evolved DNA synthesis alternatives committed to replication completion at the expense of replication fidelity, likely to ensure the timely successful chromosomal segregation in mitosis, as the existence of specialized DNA polymerases with a role in unchallenged replication suggests [151,152].

Translesion synthesis (TLS) polymerases are specialized enzymes that synthesize DNA when canonical DNA synthesis is blocked due to damaged bases that replicative DNA polymerases cannot use as templates. TLS polymerases form a DNA damage tolerance pathway that focuses on a replication bypass mechanism replacing replicative DNA polymerases when bulky lesions on the DNA impede the processive progression of DNA synthesis to proceed. The discovery of TLS polymerases was a breakthrough that revealed the existence of a DNA synthesis mechanism for tolerating DNA damage [152]. The regulation of TLS polymerases appears to be complex; for example, polymerase (Pol) eta travels with the replisome during the unchallenged S-phase, when it is SUMOylated by PIAS1 in a Rad18-dependent manner, to synthesize DNA at difficult-to-replicate sequences in animal cell lines [153]. Some TLS polymerases, such as Pol eta and Pol kappa, play a role in replicating CFSs independent of PCNA (Proliferating Cell Nuclear Antigen) ubiquitylation in both cancerous and normal mammalian cell lines [153,154,155]. However, the ability of evolutionary conserved key TLS enzymes to synthesize DNA in eukaryotes is enhanced by PCNA mono-ubiquitylation [156,157,158,159,160], as reviewed in [161]. The role of ubiquitin and SUMO modifications in DNA replication has been reviewed [162]. TLS-mediated DNA synthesis is downregulated and prevented at replication forks through PCNA deubiquitylation, most likely as a consequence of low TLS fidelity [163]. Significantly, it has been shown that PCNA ubiquitylation can operate after bulk genome replication, and, therefore, TLS DNA polymerases can effectively promote the error-prone DNA synthesis of ssDNA tracks late in G2 [164,165]. Recent evidence indicates that the processing of replicative DNA polymerase-blocking lesions is mainly a post-replicative mechanism, at least in cells that have accumulated a large amount of exogenous DNA damage [166,167]. To some extent, we can consider that TLS-mediated DNA synthesis is a DNA replication mechanism that likely emerged as a means to ensure the replication completion of under-replicated DNA tracks before mitosis. Accordingly, it has been shown that TLS DNA polymerases are involved in the replication of CFSs, non-canonical DNA structures, and difficult loci late in G2. However, this is not the case later in the cell cycle, as mitotic DNA synthesis appears to be independent from TLS [90,148,153,168,169,170,171].

### 3.2. Mitotic DNA Synthesis

As already mentioned, CFSs are widely considered to be the last regions of the human genome to be replicated [119]. Unfortunately, this contributes to their instability by preventing their complete duplication before mitosis, particularly under replication stress [172,173,174]. Under these cellular conditions, the resulting DSBs can be efficiently repaired by HR or NHEJ [175]. During HR, sister chromatids are interlinked by recombination intermediates, mainly Holliday junctions (HJs), and several endonucleases seem to be involved in their processing (reviewed in [176]). The DNA endonuclease CtIP and its partner complex MRN (Mre11/Rad50/Nbs1) promote end resection to initiate DSB repair by HR [98,177]. It has been shown that mitotic recombination at CFSs also requires CtIP and the MRN complex to prevent CFSs expression in mammalian cells. Moreover, CtIP-associated endonuclease activity is necessary for the repair of DSBs in proximity to CFSs, although it is dispensable for end resection and HR at general DSBs [178]. Thus, these factors act before mitosis to resolve the resulting entangled genomic regions and avoid DNA breakage. 

However, low levels of under-replicated and unresolved CFSs can be tolerated or not detected by the cell, and, as a consequence, the ATR/ATM checkpoint pathways are not activated. Therefore, cells do not arrest in the S-phase to complete replication, and under-replicated CFSs persist as late as early mitosis [131,135,179]. Under this situation, it is known that mitotic cells activate special mechanisms to manage these under-replicated structures beyond the S-phase [90]. Firstly, FANCD2, the main mark of CFSs in mitosis, guides the structure-selective endonuclease MUS81-EME1 (catalytic and regulatory subunits, respectively) and its platform protein SLX4 to the CFSs to process these under-replicated or unresolved DNA structures during the late G2 phase and early mitosis [135,144,149,169,179,180,181,182]. Next, mitotic cells also activate unscheduled DNA synthesis to complete the replication of those processed FANCD2-marked regions [144] (Figure 1). In this way, the MUS81-EME1-mediated DSBs induce a non-canonical POLD3-dependent DNA synthesis pathway across prophase and early prometaphase, which has been termed MiDAS (for mitotic DNA synthesis) [144]. From a mechanistic point of view, it has been suggested that MiDAS is a form of break-induced replication (BIR), a conservative recombination-dependent DNA replication process that utilizes a single-ended DSB to restart replication at broken or collapsed forks in a Rad52-dependent manner. In contrast to the canonical HR pathway, BIR is an error-prone type of HR that can lead to genomic instability [183,184,185,186]. Further work is needed to better understand the link between MiDAS and BIR mechanisms.

It has been shown that the cell cycle machinery itself regulates MiDAS. Accordingly, Cdk1 kinase, the major regulator of mitosis, phosphorylates EME1 and SLX4 at the onset of mitosis to promote their interaction to form an SLX-MUS complex (SLX1-SLX4-MUS81-EME1) [82,187] (Table 1). This mechanism is similar to the one described for budding yeast, in which the orthologues Mus81-Mms4 are hyperactivated at the G2/M transition by Cdc28/Cdk1 and Cdc5 (polo-like kinase 1 or Plk1 in vertebrates) [81,188]. Of particular note, the CDK inhibitor Wee1 kinase, a negative regulator of the entry into mitosis, also interacts with MUS81-EME1 [189]. Wee1 is important for the control of replication fork speed during the S-phase (Figure 1) and its depletion leads to DNA replication fork slow down, as well as DDR that is triggered in a MUS81-EME1-dependent manner [189]. Thus, while the MUS81-EME1 complex has low activity during interphase, probably due to Wee1 interaction, binding to SLX4 increases the resolvase activity in mitosis. This association has been shown to be important for the recruitment of the MUS81-EME1 complex to CFS under-replicated loci [144,149,182], whose nuclease activity consequently promotes POLD3-dependent DNA synthesis at CFSs in mitosis, as previously mentioned [144,190]. ERCC1, another structure-selective endonuclease that forms a complex with XPF, also seems to be involved in processing unresolved CFSs at mitosis [149]. In fact, the lack of MUS81 and ERCC1 causes an increased level of unprocessed CFS intermediates that escape the faithful sister chromatid disjunctions [149,182]. However, considering new evidences suggesting that MUS81 is implicated in the resolution of termination intermediates generated by converging HR-restarted forks [191], the mechanistic details by which these factors act in CFSs processing remain to be fully elucidated.

Regarding the molecular mechanism involved in MiDAS, additional experimental data have raised the question of whether other endonucleases or other factors are also implicated. Work on yeast has shown that the BIR pathway can still be activated in the absence of Mus81 [192]. Moreover, it has been shown that chicken DT40 cells lack a MUS81 orthologue [193] and that, in these cells, mitotic SLX4 foci recruitment can be FANCD2 independent [150]. Interestingly, the topoisomerase IIα-binding protein 1 (TopBP1) has been shown to be the protein responsible for mediating SLX4 recruitment and mitotic DNA synthesis [150]. 

Moreover, recent studies have expanded our understanding of the MiDAS pathway as a link between DNA replication and mitosis. These works have found that the RING E3 ubiquitin ligase TRAIP is an important regulator of the processing of incomplete DNA replication during mitosis in metazoan. Thus, TRAIP drives replisome disassembly at those under-replicated CFS loci, thereby allowing access to replication fork for the specific factors in charge for mitotic DNA synthesis [194]. In addition, the activity of TRAIP during mitosis seems to be regulated by Cdk1-Cyclin B1 complexes [56,194,195], which control the accurate timing of mitosis and also regulate the switch from conventional DNA replication to MiDAS machineries (Table 1). Together, these data suggest that the mechanisms of MiDAS have only just started to be unraveled.

When MiDAS fails, the persistence of under-replicated CFSs in late mitosis results in the formation of ultrafine bridges (UFBs) during anaphase–telophase [145] (Figure 1). UFBs are characterized by the persistence of FANCD2, which is not bound across the bridge but to its ends [135,179]. Moreover, other proteins, such as the Plk1-interacting checkpoint helicase (PICH), the Bloom syndrome helicase (BLM), and RIF1, also coat these UFBs [149,182,196,197,198,199]. Although the exact nature of the structure underlying these UFBs, as well as the molecular mechanisms involved in their resolution, are still poorly understood, it is thought that the coordinated activity of the mentioned proteins is required for UFB resolution and sister chromosome disjunction at this final cell cycle point [145]. Thus, the majority of these UFBs are resolved during late anaphase or telophase. 

The persistence of under-replicated CFSs by the end of mitosis has been associated with many different genomic instability events, such as DSBs, potential chromosome rearrangement [200], and chromosome mis-segregation, which can cause binucleation [150] or micronuclei formation, potentially leading to the mutagenic event known as chromothripsis (a tumorigenic mechanism characterized by chromosome fragmentation that leads to thousands of clustered chromosomal rearrangements in small genomic regions in a single event) [135,179,201].

Unresolved UFBs can lead to DNA breakage and the ATM-dependent formation of 53BP1 (p53 binding protein 1) positive foci, replacing the FANCD2 ones. 53BP1 is a DNA repair marker that associates with DSBs, under-replicated DNA, or unresolved DNA structures at the end of mitosis. The 53BP1 bodies have been thought to promote DNA repair through NHEJ in G1 or to shield DNA lesions until the next S-phase, when they can be repaired by HR mechanisms [149,182,202,203,204,205]. Recent work by Spies et al. has provided new data indicating that if under-replicated DNA is not repaired by MiDAS in mitosis, it is transmitted to the daughter cells, where it is englobed by 53BP1 nuclear bodies during the beginning of the new cell cycle. By recruiting Rif1, these 53PB1 nuclear bodies ensure that the inherited unreplicated loci will be replicated only in the late S-phase in a RAD52-mediated repair way. If 53PBP1 or its partners fail, RAD51 will promote repair leading to aberrant recombination structures and subsequent chromosome aberrations in the following mitosis. Therefore, it would perhaps be more accurate to say that MiDAS represents the penultimate chance to finish incomplete replication and that this 53BP1-dependent mechanism may be the last chance [206]. Moreover, 53BP1 bodies can drive the cell to enter into a quiescent state in a p53 depending manner [207]. Collectively, all these data support the importance of unscheduled DNA synthesis in late G2 to early mitosis to avoid genomic instability.

Although CFSs are considered the main genomic sites for mitotic DNA synthesis [144], similar events have also been observed at telomeres [208,209], another difficult-to-replicate genomic region [116]. Whether or not MiDAS at CFSs and telomeres share the same mechanisms, and whether or not unscheduled mitotic DNA synthesis is exclusive of these two regions, remain unanswered questions. 

The existence of MiDAS, a special DNA synthesis process accomplished outside of the S-phase, somehow implies that proteins which are characteristic of S-phase also accommodate working outside this cell cycle stage, particularly during mitosis. Thus, FANCD2 is involved in the repair of interstrand crosslinks facilitating CFS replication completion and stability before mitosis [134], extending its function beyond the S-phase, and participating in unscheduled DNA synthesis in late G2 to early mitosis, as already mentioned. Although the precise function of FANCD2 in MiDAS is currently poorly understood, it has been shown that the depletion of TRAIP impairs FANCD2 foci formation in mitosis, as well as mitotic DNA replication [194]. Future studies will be needed to determine if this observation is just a correlation or if FANCD2 indeed plays an active role in MiDAS. Moreover, if recovery from replication stress is unsuccessful or incomplete, FANCD2 remains bound to CFSs during mitosis, even up to the telophase [135,179] to likely promote resolution of the generated UFBs, limiting chromosome mis-segregation [135].

In addition to FANDC2, other factors, such as PICH, BLM, and RIF1, involved in DNA replication and DDR, also have an important impact on MiDAS [149,182,196,197,198]. RIF1 is a protein with important roles in DNA replication timing and DSB repair processes, among others, which is recruited to FANCD2-positive UFBs to participate in their resolution in the anaphase [198,199]. Another example is the already mentioned TopBP1, a multifunctional protein involved in processes such as transcriptional regulation, DNA replication, ATR-dependent checkpoint pathway, and DNA repair, which also exerts its action beyond the S-phase, thereby contributing to supporting unscheduled DNA synthesis [150]. 

Collectively, under replication stress conditions, incomplete DNA replication, particularly at CFSs, represents a serious danger for chromosome segregation. The processing of these under-replicated intermediates and the following MiDAS constitute an important feature, where a plethora of proteins, initially framed in the S-phase processes, participate to resolve these abnormalities in mitosis, contributing to chromosome stability maintenance. Although the role of these proteins in this process has started to be characterized, we still do not know how they are regulated, how they interact with each other, how they associate to mitotic chromatin, or what type of DNA structures they act on. Moreover, other still-unknown proteins most likely participate in MiDAS. Their identification will also provide important clues to the pathways involved in both the unscheduled DNA synthesis in late G2 to early mitosis and the processes that cells activate to resolve UFBs at the end of mitosis.

## 4. DNA Damage in Mitosis

### 4.1. Mitotic DDR

Cells have evolved mechanisms to preserve genomic integrity throughout the cell division cycle. Thus, the maintenance of genomic stability depends on how DNA-associated processes, notably replication, transcription, and mitotic segregation, occur throughout the cell cycle. The decision to enter mitosis mainly depends on the full activation of the mitotic Cdk1 kinase. This situation is modulated by regulatory mechanisms that interface with other mitotic kinases and checkpoint pathways that safeguard the integrity of the genome before entering mitosis [8,9,10,210]. For example, ATM/ATR checkpoint kinases lead to the degradation of the Cdc25 phosphatases with the consequent inactivation of Cdk1 and cell cycle arrest to block mitosis when DNA repair occurs. It is also wellknown that the mitotic kinase Plk1 is a target of the DNA damage checkpoint both in budding yeast and human cells [211,212,213] (Figure 1). In response to DNA damage during G2, Plk1 is inhibited in an ATM/ATR-dependent and p53-independent manner to induce cell cycle arrest at G2/M [213,214,215,216]. In addition, Plk1 regulates cell cycle resumption upon DNA damage in G2 by targeting some inhibitors of Cdk1 activity [53,217,218]. The CDK activity itself seems to be required for an efficient DDR [78,219,220], which confirms the close connection between the cell division cycle and DDR mechanisms. 

Once cells enter mitosis, the spindle assembly checkpoint (SAC), the main checkpoint in mitosis, becomes active and keeps acting until each chromosome is properly attached to the mitotic spindle, ensuring faithful segregation of the chromosomes [16]. The proteins BubR1, Bub3, and Mad2 form the mitotic checkpoint complex (MCC), the main component of the SAC, which inhibits APC/C by binding to its activator Cdc20. The Cdk1 activity has a central role in SAC activation through phosphorylation of some of the proteins involved in the SAC. Thus, Cdk1 phosphorylates the key organizer of the SAC signaling cascade, monopolar spindle 1 (Mps1) kinase, to allow its recruitment to kinetochores, from where Mps1 leads the consequent recruitment of additional SAC components [72]. The Cdk1-mediated phosphorylation of the Bub1 mitotic checkpoint kinase, is also critical for SAC activation in human cells [71]; and the Cdk1 phosphorylated form of the APC/C activator and the MCC component Cdc20 have been shown to have more affinity for MCC than for the APC/C complex [73] (Table 1). Moreover, the finding that cyclin B1 is located to unattached kinetochores, where SAC initiates, also confirms the importance of Cdk1-cyclin B1 in SAC activity [221,222,223]. APC/C promotes anaphase through the degradation of securin, which keeps sister chromatids together. Once each chromosome is located at the metaphase plate, SAC is satisfied, Cdc20 is released from MCC, and APC/C activity initiates the anaphase [16].

Until recently, it was considered that once cells are committed to mitosis, the only functional checkpoint is SAC and also that DDR becomes irrelevant because the core DNA damage repair machineries are suppressed, thus, preventing chromosome breaks [55]. Mitotic cells can face DNA damage (for example, DSBs) as a result of persisting interphase errors or due to the exposure to DNA damaging agents already in mitosis. In fact, mitosis is a well-known therapeutic target, and many chemotherapeutic drugs induce prolonged mitotic arrest with the consequent accumulation of DNA damage and cell death as the preferred final outcome. There is evidence of a mitotic DDR that senses this damage and activates checkpoint pathways that are similar, at least in their early stages, to those of the conventional known interphase ones (Figure 1). Thus, mitotic DSBs show γH2AX modification and lead to the recruitment of DDR factors, such as the MRN complex and the mediator of DNA damage checkpoint 1 (MDC1), pointing to where the damage is and activating ATM, ATR, and DNA-PK, in a similar way to what occurs in the interphase [214,215,224,225,226,227,228,229,230,231].

Telomeres are particularly sensitive to prolonged mitotic stress and are considered the regions where the DDR is initiated. The caspase-dependent cleavage of proteins, such as the shelterin complex component TRF2, causes telomere protection loss with the consequent DNA strand breaks and the activation of ATM and DNA-PK kinases [232,233,234]. A recent work pointed out that the cell death induced by a high level of replication stress occurs predominantly in mitosis through telomere deprotection and DDR activation, a process in which the ATM kinase is also involved [235]. Moreover, there is evidence that the mitotic DDR exists even in the absence of DNA damage to coordinate spindle assembly and kinetochore/microtubule attachment. Thus, the misregulation of DDR elements during mitosis alters the faithful chromosome segregation process [236].

However, several lines of evidence show that mitotic DDR works differently than the DDR activated during interphase. For instance, in contrast to DDR during the G2 phase, where Plk1 is inactivated to prevent mitotic entry [213], mitotic DNA damage seems to induce increased levels of Plk1 activation (Figure 1), which is necessary, at least in part, to mediate the effects of the DDR on kinetochore-microtubule attachments and chromosome segregation [237]. Another example arises when comparing the DDR response to telomere deprotection, due to the spontaneous reduction or loss of shelterin complex during cellular aging, in which there are no DNA breaks, to the genomic DDR induced by a number of DNA damaging agents, such as replication poisons or ionizing irradiation. Both mechanisms activate the ATM kinase, but while genomic breaks induce the canonical DDR signaling pathway, telomere deprotection does not activate the ATM-downstream factor, Chk2. Consequently, the G2/M checkpoint is not activated, the cells pass through and exit mitosis with deprotected telomeres, and arrest occurs in the next G1 in a p53-dependent manner [232]. However, Chk2 can be activated independently of ATM by the phosphorylation of other mitotic kinases, such as DNA-PK and Plk1 [236,238,239]. Moreover, additional evidence indicates that, in response to ionizing radiation, DDR-mediated Chk2 activation can even occur through the phosphorylation of non-canonical residues [228,237]. Taken together, these data indicate that aside from the shared DDR initial factors, downstream mitotic DDR signaling cascades are quite different from those working during the interphase. 

Although the SAC differs from any known DNA damage checkpoint, recent data suggest that there is some crosstalk between the mitotic DDR and the SAC. In most cases, these findings reflect the interactions observed among proteins involved in each of the two pathways. Thus, BubR1 kinase, a component of the MCC, interacts with MDC1 and Plk1 during mitosis. In turn, MDC1, together with ATM and γH2AX, seems to be required for kinetochore localization of the MCC proteins, Mad2 and Cdc20 [240]. Moreover, several studies have reported that the DDR kinases, ATM and ATR, play a role in SAC control, both in cells exposed to DNA damage and in unperturbed cells through the regulation of MCC formation or the recruitment of MDC1 to kinetochores [240,241]. Thus, cells respond to mitotic damage slowing down the passage through mitosis by stabilizing the kinetochore and activating the SAC through ATM and ATR checkpoint kinase activity (see [242] for details) (Figure 1). The molecular details of how mitotic DDR keeps an active SAC remain unknown.

### 4.2. Repair in Mitosis

Despite the aforementioned evidence on the existence of a specific mitotic DDR, it is thought that DDR does not activate a full DNA repair program [150,237]. It has been shown that Cdk1 inhibitory phosphorylation of DNA repair factors, such as 53BP1 and the E3 ligase RING-finger protein 8 (RNF8), required to initiate repair mechanisms, blocks the recruitment of these proteins to DSB foci, impairing the activation of canonical DNA repair mechanisms in mitosis [55,228,243] (Table 1). Thus, by averting this Cdk1-dependent inhibition of the DNA repair machinery or inducing ectopic DNA repair, cells exhibited increased rates of sister telomere fusions and chromosome mis-segregation [55,237]. In the same way, the observed Plk1 activation in response to DNA damage in mitosis could also be involved in blocking this repair cascade at the level of RNF8 recruitment, due to additional inhibitory phosphorylation of RNF8 and 53BP1 proteins [55]. Another block to mitotic NHEJ mechanisms involves the regulation of XRCC4, a regulatory subunit of the NHEJ ligase IV complex, which is also inhibited during mitosis by CDK and Plk1 activities causing suppression of DSB repair. A non-phosphorylatable XRCC4 mutant increases both the efficiency of DSB repair in mitosis and the formation of anaphase bridges [79]. Moreover, the proteins involved in the HR repair pathway, including BRCA2 and RPA, are also inhibited by mitotic Cdk1 phosphorylation, which contributes to blocking the RAD51 filament formation needed for DSB repair [229] (Table 1). This evidence supports the fact that high Cdk1 activity avoids DNA repair during mitosis. Recently, it has been observed that during mitosis, MDC1 recruits TopBP1 to mitotic DSBs to form filamentous structures that allow the proper segregation of unrepaired chromosomes [226]. Therefore, the initial activation of mitotic DDR could provide structural support for marking damaged DNA until the next G1 phase, when NHEJ-mediated repair mechanisms take place. The existing evidence indicates that cells silence DNA repair mechanisms during mitosis to prevent chromosomal instability or mitotic catastrophe. When, and exactly how, mitotic DNA repair is stopped in mitosis is not completely known. 

Sister chromatid non-disjunction, manifested in late mitosis as UFBs, are a potential source of genome instability [244]. Strictly speaking, mitotic DNA damage repair deals with unfinished S-phase processes. Beyond the already described unscheduled POLD3-dependent DNA synthesis at unreplicated CFSs [144] and telomeric regions [209,245] during the early prophase, mitotic cells also activate pathways to deal with a type of joint DNA molecules, the Holliday junction (HJ), which constitute another class of liability for chromosome segregation. The persistence of HJs in mitosis, a consequence of failed recombination events during the S- and G2 phases, seems to induce a mitotic-specific repair mechanism triggered by the Cdk1-dependent interaction between SLX1-SLX4 and MUS81-EME1 endonuclease to act on HJs [82]. The resolvase GEN1, activated at the entry into mitosis, appears to also be involved in the processing of HJ, but through a parallel pathway [83,246]. There are four major types of UFBs depending on their origin and underlying structure as follows: Replication stress can cause FS-UFBs, characterized by FANCD2 twin foci, specially at CFSs, and mitotic DNA synthesis; centromeric regions may be bound by double-stranded catenanes creating C-UFS that are resolved by topoisomerase II; fork stalling at telomeres or telomeric end fusion create T-UFBs, as well as R-UFBs defined by rDNA and also modulated by Topo II [135,179,197,247,248,249]. The FA pathway collaborates with BLM in the resolution of FS-UFBs by targeting it to non-centromeric bridges, whereas BLM, but not FA proteins, is required to resolve C-UFBs [135,136]. Interestingly, it has been recently shown that there is a fifth type of UFB that arises from unresolved recombination intermediates in cells deficient in the nucleases GEN1 and MUS81. These intermediates are named homologous recombination ultrafine bridges (HR-UFBs) and are different from FS-UFBs and C-UFBs. Unlike FS-UFBs, HR-UFBs are not induced by replication stress, and most of them are not associated with FANCD2 foci. HR-UFBs are converted into ssDNA in a PICH and BLM dependent process, generating ssDNA that are coated by RPA and break at the end of mitosis. As a consequence, the DDR is activated in the next cell cycle causing G2 arrest, and NHEJ gives rise to chromosome fusions, which will eventually lead to massive cell death. It seems that BLM and PICH are part of a common mechanism that processes HR-UFBs, FS-UFBs, and C-UFBs [145]. Again, the mitotic activation of these resolution pathways constitutes a last-ditch attempt to resolve the recombination intermediates that escape S-phase repair mechanisms.

## 5. Concluding Remarks 

Mitosis constitutes the final and most dramatic phase of the cell cycle, where cells orchestrate crucial changes in multiple cellular components and activate intricately signaling pathways to govern the dynamic reorganization of their cellular structure. The final goal of mitosis is the division of duplicated chromosomes resulting in two genetically identical daughter cells. The failure of faithful chromosome segregation often results in genetic instability, which culminates either in cell death or in cancer [250]. The well-known DDR signaling pathways, activated during interphase, work to safeguard the genome during the G1, S-, and G2 phases of the cell cycle, but mitotic cells can still encounter DNA lesions as a result of persistent replication deficiencies, unrepaired pre-mitotic damage, and true mitotic DNA damage. Recent evidence indicates that a DDR also exists in mitosis to deal with this damage, although its role is only beginning to be known. Mitotic DDR pathways seem to extensively differ from the interphase ones, and DNA repair mechanisms are not yet fully understood. Thus, although numerous works support the idea that DNA repair is largely suppressed in mitosis [55,228], recent studies suggest otherwise. Mitotic-specific repair events have been identified, including those focused on the resolution of under-replicated CFSs and telomeric regions, as well as the resolution of persistent recombination intermediates to minimize the associated genetic instability. It is known that, in response to mitotic DSBs, the initial steps of the DDR are activated to mark DNA lesions in a similar way to what happens for subsequent repair. However, the signaling cascade is inhibited downstream, and the recruitment of early DDR elements to mitotic DSBs does not lead to repair but instead provides structural support until the next cell cycle to be repaired. Silencing DSB repair in normal cells during mitosis seems to prevent the structural chromosomal instability caused by potentially aberrant NHEJ or the fusion of deprotected telomeres [55,237]. Why different DNA lesions are processed differently during mitosis is an interesting open question. Importantly, because Cdk1 avoids general mitotic DNA repair processes through the direct inhibitory phosphorylation of diverse repair factors, and because Cdk1 is also necessary to mitotic DNA synthesis at CFSs through direct phosphorylation of proteins involved in this process, the importance of this kinase in genomic stability maintenance is highlighted again. How Cdk1 is regulated to accomplish inhibition of DSB repair and activation of some specific mitotic repair mechanisms is not well understood. Many proteins involved in DNA replication during the S-phase are also required for the proper resolution of under-replicated CFS intermediates during mitosis. All these works show the importance of the cell cycle in the regulation of DDR, especially those mechanisms involved in DNA repair. In the coming years, it is likely that how cells manage DNA lesions or abnormal DNA structures during the different cell cycle phases, and especially during mitosis, will be better understood.

## Figures and Tables

**Table 1:** A list of the main cellular processes involved in the maintenance of genomic stability along the cell division cycle and the role of cyclin-dependent kinases in them. Note that the proteins listed can be direct or indirect CDK substrates. We refer the reader to the indicated references for specific and detailed information regarding each direct or indirect CDK substrate.

Cell Cycle Processes Related to Genomic Stability Maintenance and Their CDK-Dependent Regulation		
Process	Substrate(s)	Outcome of CDK Phosphorylation
Replication origin licensing	MCMs Cdc6 Cdt1 ORC	Impairs loading on chromatin and DNA synthesis initiation in G1 [35] Regulates stabilization and subcellular localization during G1 [36,37] Induces degradation at G1/S [38,39] Inhibits helicase loading [3,40,41]
Replication initiation, G1/S-phase transition	MCMs GINS TRESLIN Rb Cdc7	Activation of MCMs-Cdc45 helicase activity in the S-phase [42,43] Recruitment to chromatin [44] Promotion of DNA replication and association to TopBP1 [45,46,47] Rb inactivation and induction of the S-phase genes transcription in late G1 [48,49,50] Kinase inactivation [51]
DNA repair and G2 DNA damage checkpoint recovery (continues on the next page)	53BP1 RNF8	Stable binding to Plk1 to silence DNA damage checkpoint and promote entry into mitosis [52,53]; abolition of 53BP1 binding to DSB-flanking chromatin in mitosis [54,55] Impairs RNF8-MDC1 association in mitosis [55]
DNA repair and G2 DNA damage checkpoint recovery	TRAIP ATRIP FANCC/FANCG Rad9 RPA Srs2 helicase FOXO1 EXO1	Activation to promote CMG unloading at stalled replication forks [56] G2/M checkpoint maintenance [57] FA core complex chromatin localization in G2/M [58,59] Interaction with TopBP1 [60] Regulation of DNA repair pathway [61] DSB repair by synthesis-dependent strand annealing [62] Inhibition of FOXO1 induced-apoptosis in presence of DNA damage [63] Regulation of DNA resection and repair pathway choice [64]
Mitotic entry	FOXM1 Plk1 Wee1 Myt1 Cdc25A/B/C	Activation of mitotic transcriptional program [65,66] Activation [67] Degradation [68] Inhibition [69] Activation [70]
Spindle Assembly Checkpoint (SAC)	Mps1/Bub1 Cdc20	SAC activation [71,72] APC/C inhibition [73]
Homologous recombination (HR)	BRCA2 RECQL4 CtIP Crb2	Block BRCA2-RAD51 interactions as cells approach mitosis [74] Enhance interaction MRE11/RECQL4 and RECQL4 recruitment to DSBs and stimulated activity [75] Interaction with BRCA1 and NbsI [76,77] Resolution of HR intermediates [78]
Non-Homologous End Joining (NHEJ)	XRCC4	NHEJ suppression in mitosis [79]
MiDAS	RECQ5	Processing of CFSs by Mus81-EME1 in mitosis [80]
Holliday Junction resolution	SLX4/Mus81 Gen1	Formation of the SLX-MUS complex and activation [81,82] Unknown [83]
Sister chromatid junction resolution	TopBP1-SLX4	Promotes TopBP1-SLX4 interaction [84,85]

CMG: CDC45, MCM2-7, GINS complex; GINS: protein complex essential for DNA replication formed by Sld5, Psf1, Psf2, and Psf3 subunits; TRAIP: E3 ubiquitin ligase TRAIP; MDC1: mediator of DNA damage checkpoint 1; MiDAS: mitotic DNA synthesis.
